# Artificial intelligence and machine learning in neurogenic lower urinary tract dysfunction and spinal cord injury: current status, emerging technologies, and ethical perspectives

**DOI:** 10.3389/fruro.2026.1931923

**Published:** 2026-08-07

**Authors:** Patrick M. F. Levien, Jürgen Pannek

**Affiliations:** 1 Neuro-Urology, Swiss Paraplegic Center, Nottwil, Switzerland; 2 Department of Urology, Inselspital, Bern University Hospital, University of Bern, Bern, Switzerland

**Keywords:** artificial intelligence, autonomic dysreflexia, clinical decision support, closed-loop neuromodulation, deep learning, detrusor-sphincter dyssynergia, digital twin, health equity

## Abstract

Artificial intelligence (AI) and machine learning are increasingly applied across urology, with particular promise for the management of neurogenic lower urinary tract dysfunction (NLUTD) in individuals with spinal cord injury (SCI). There is also particular promise in the management of NLUTD, a complex, lifelong condition affecting individuals with SCI and also occurring in the context of multiple sclerosis, Parkinson’s disease, and other neurological disorders. This article provides a comprehensive analysis of AI’s current and emerging role in neuro-urology, with dedicated focus on the specific challenges and opportunities presented by NLUTD and SCI. We review the epidemiological and clinical burden of neurogenic bladder dysfunction. We further examine AI applications in uro-oncology, functional urology, digital pathology, and robotic surgery, integrating these with the specialized perspective of neuro-urology. The particular vulnerability of individuals with SCI to algorithmic bias, access inequity, and assistive technology dependency is critically discussed. We identified five key domains where AI may transform care for patients with NLUTD: automated urodynamic interpretation including detection of detrusor overactivity and detrusor-sphincter dyssynergia, predictive risk stratification for renal deterioration, closed-loop neuromodulation, remote digital monitoring, and AI-assisted rehabilitation support. Alongside the substantial opportunities, we articulate the ethical, moral, and societal responsibilities that accompany AI integration in this population, emphasizing that patients with SCI and NLUTD deserve not merely inclusion in AI development but active partnership as co-designers of the technologies that will shape their lives.

## 1 Introduction

The annual publication rate for AI research in healthcare has shown exponential growth, particularly since 2014. Between 2000 and 2022, the number of AI-related publications in PubMed increased 36-fold to a total of 307,701 publications (1). The average annual growth rate was 17.02% since 1995, but accelerated to 45.15% between 2014 and 2019 (2). A similar trend can be observed in urology, with a particularly sharp increase over the last ten years (3).

Especially artificial intelligence (AI) and machine learning (ML) are reshaping the practice of urology at a pace that few specialties have previously experienced. In 2019, a systematic review of 111 studies on AI in urology showed that, in 71.8% of the studies, AI approaches were superior to conventional statistical methods in terms of diagnosis and prognosis (3). By 2026, across the spectrum of diagnostic imaging, digital pathology, robotic surgery, stone therapy, clinical decision support, nursing and drug development, AI-driven tools are increasingly entering clinical practice with a growing number receiving regulatory approval and accumulating evidence of patient benefit (4). Within this broader transformation, patients with neurogenic lower urinary tract dysfunction (NLUTD) following spinal cord injury (SCI) or other conditions stand also to gain from AI’s capabilities while simultaneously facing distinctive risks from its limitations (5–8).

NLUTD represents a lifelong challenge for the estimated 27 million people worldwide living with SCI, and for the much larger population of individuals with neurologically mediated NLUTD arising from multiple sclerosis (MS), Parkinson’s disease, spina bifida, stroke, or diabetic neuropathy. Urological complications, including recurrent urinary tract infections (UTIs), upper urinary tract deterioration including renal failure, vesicoureteral reflux, or bladder tumors, are the leading causes of preventable mortality and morbidity in long-term SCI survivors. Through systematic urodynamic surveillance and guideline-driven management, renal failure as leading cause of death in SCI has been substantially reduced (9). However, treatment failure rates and adherence to drug treatment remain far from perfect with approximately 40-60% for oral antimuscarinics and 25-35% for intradetrusor botulinum toxin injections in neurogenic detrusor overactivity (10). To manage NLUTD successfully, both objective risk factors and quality of life (QoL) must be considered, and ideally management is based on participatory decision-making. As this approach requires longitudinal, individualized, and data-intensive clinical care, AI is uniquely positioned to support.

In this article, we address five central questions:

1. What is the current role of AI in urological diagnostics and therapy?
2. Which AI tools are already clinically deployed, and with what efficacy?
3. Are there technologies actively being developed, particularly for neuro-urological applications?
4. What are the opportunities and risks of AI in urology, with attention to the specific vulnerabilities of patients with NLUTD?
5. Which ethical, moral, and societal implications arise from AI adoption in this context?

Our perspective is that AI in urology offers transformative potential that is especially meaningful for patients with NLUTD, and that realizing this potential demands inclusion, equity, rigorous validation, and unwavering patient-centeredness.

## 2 Neurogenic lower urinary tract dysfunction: clinical burden and the case for AI

NLUTD can arise from neurological lesions at any level of the nervous system. Each neurological level generates a characteristic but variable pattern of NLUTD, and individual variability is substantial even within the same diagnostic category. Suprasacral spinal lesions typically produce detrusor overactivity (DO), often combined with detrusor-sphincter dyssynergia (DSD), a pattern that generates high intravesical pressures threatening upper urinary tract function. Infrasacral lesions more commonly cause detrusor underactivity (DUA) or an acontractile bladder with impaired emptying.

The urological stakes in SCI are particularly high. A landmark prospective study of patients with acute traumatic SCI demonstrated that 90 out of 97 patients (90%) showed one or more unfavorable urodynamic parameters within the first year after injury. Detrusor overactivity with DSD was present in 88% of cases, maximum storage detrusor pressure of 40 cmH2O or higher in 39%, and vesicoureteral reflux in 7% of cases. Critically, the clinical symptoms of patients with SCI correlate poorly with their urodynamic findings, meaning that objective urodynamic assessment is indispensable and cannot be replaced by symptom reporting (9). This dissociation between subjective symptoms and objective bladder behavior is a challenge for AI systems intended to predict or monitor NLUTD, and it represents a key reason why AI tools must be validated specifically in neurological populations rather than extrapolated from non-neurological cohorts (5, 11).

The complexity of NLUTD management is compounded by its dynamic nature: NLUTD evolves over time, requiring repeated urodynamic reassessment, frequent therapy adjustments, and sustained longitudinal monitoring. The recommended EAU follow-up schedule after SCI includes urodynamic investigations at 1, 3, 6, and 12 months after injury, and then annually thereafter, a resource-intensive requirement that strains neuro-urological services globally. AI has the potential to substantially ease this burden through automated urodynamic interpretation, predictive risk stratification, and remote monitoring, while simultaneously improving the precision and personalization of care.

## 3 Current status and trajectory of AI in urology

AI applications in urology are growing exponentially. The growth rates in various urological sectors between 2004–2013 and 2014–2023 were particularly striking with +476% in clinical (non-surgical) applications, + 616% in clinical (surgical) applications and +185% in training applications (12). AI technologies span diagnostic imaging, digital pathology, functional diagnostics, surgical assistance, and clinical decision support (**Table 1**). Within neuro-urology, the most clinically mature applications involve automated urodynamic signal interpretation and predictive modeling for treatment outcomes.

**Table 1:** Overview of key AI applications relevant in neuro-urology at March 2026.

Domain	AI application/status
AI enhanced robotic surgery	- Stone Therapy by robot-assisted ultrasound-guided PCNL (13, 14) - Aspects of surgical training, enhancement of surgical experience, preparations for autonomous robotic surgery (15, 16)
Bladder cancer	- AI cystoscopy (17) - Computer-aided bladder cancer diagnostics (imaging) (17) - AI Applications in bladder cancer (17) o prediction o recurrence prediction o Chemotherapy response prediction - AI urine biomarker analysis and pathology (18)
Urodynamics	ML models for Detrusor Overactivity, Detrusor Underactivity, Bladder Outlet Obstruction detection (11, 19); Large Language Models in automated interpretation (20, 21) Automated Evaluation of Urodynamics by Local linear Models (8) Classification of results of uroflowmetry (22)
SCI risk stratification	Classification of Bladder Dysfunction severity (23, 24) ML models predicting upper urinary tract damage (23); NB incidence prediction models
Diagnostic and therapy of stones	ML and DL for stone therapy (25, 26)
Wearable/remote monitoring (27)	NIRS transcutaneous optical bladder monitoring (28); Virtual Bladder strain sensors (29) Real-time closed-loop neuromodulation against Detrusor overactivity (30)
Neuromodulation (SNM)	AI-assisted closed-loop sacral neuromodulation using dorsal root ganglia (31) Adaptive stimulation for neurogenic Detrusor Overactivity (32)
UTI	ML models for multidrug-resistant Urinary Tract Infection prediction in SCI patients (26) Improvement of detection and diagnosis (e.g. Schistosoma) (33)
AI in healthcare communication, education, organization and rehabilitation	AI-solutions in healthcare (34) Virtual rehabilitation assistants (general) (35)

AI Artificial Intelligence, ML Machine learning, DL Deep Learning, LLM Large language model; UTI, urinary tract infection.

The broader urological AI landscape provides important context for urological applications. AI-augmented multiparametric MRI (mpMRI) interpretation for prostate cancer detection demonstrated that AI system detected 6 - 8% more cases with Gleason grade group 2 or greater cancers at the same specificity of non-AI-augmented detection (36). It has been projected that by 2030 AI could automate a large proportion of routine pathological quantification with high sensitivity, specificity and concordance, although such forecasts remain speculative (18). Furthermore, AI-enhanced cystoscopy systems have demonstrated detection accuracy of 0.939 in multicenter studies of bladder cancer (37). However, the significance of this for bladder cancer in patients with NLUTD remains unclear, as in this patient group, diagnosis without histologic examination is more difficult than in persons without NLUTD (38). In sum, AI can deliver clinically significant improvements in urological diagnostics.

For AI in neuro-urology specifically, the evidence base is younger but expanding rapidly. The focus here is primarily on three areas of application: urodynamic testing, the diagnosis of functional disorders, and the prediction of treatment response (39). In addition, AI applications for phenotyping, classifying the severity of NLUTD, and developing prognostic models for damage to and regeneration of the upper urinary tract are already attracting increasing attention (6, 40). Especially, AI-guided prediction of treatment response would be valuable for patients with NLUTD, where the high medical treatment failure rates create substantial clinical uncertainty about optimal therapeutic sequencing.

## 4 AI tools currently in clinical use at neuro-urological patients

### 4.1 Urodynamics: the cornerstone of neuro-urological diagnosis

Urodynamic investigation (UDI) remains the gold standard for characterizing NLUTD and guiding treatment decisions. It is currently irreplaceable in neurological populations because symptom reporting is unreliable (41). The EAU Guidelines on Neuro-Urology mandate regular UDI for all patients with SCI and other forms of NLUTD. Although standards exist for equipment, training and good urodynamic practice (GUP), urodynamic interpretation still suffers from inter-observer variability and low inter-rater reliability (42). AI can offer a compelling solution to this variability.

Several machine learning and deep learning systems for automated urodynamic interpretation have been introduced. In 2022, Hobbs et al. developed, on the basis of 1154 pediatric studies following the ICCS Guidelines and the UMPIRE consensus statement a proof-of-concept machine learning pipeline with an accuracy of 81,35% for detecting detrusor overactivity (7). A real-time urodynamic signal recognition system developed by Liu et al. has been published in 2025. This system is using a Yolov5l-based deep learning architecture trained on 2 cohorts of adult patients with NLUTD. The cohorts consisted of 300 patients with 1960 images and 100 patients with 695 images (43). The system demonstrated reliable recognition with an accuracy up to 95% of six key urodynamic signal classes, including DO waves, rectal peristalsis, abdominal muscle contractions, and three grades of cough wave quality. This is enabling a further step to automated quality control of the urodynamic examination in real time.

A deep neural network combining ResNet-50 with short-time Fourier transform (STFT) analysis, validated in 1,949 patients across two centers, achieved an accuracy of 88,6% for bladder outlet obstruction (BOO) and of 85,4% for detrusor underactivity (DUA) in the validation set (44). These results suggest that, although still imperfect, AI-based interpretation tools are approaching expert level accuracy for selected urodynamic diagnoses.

The clinical significance of automated urodynamic interpretation for patients with NLUTD extends beyond accuracy. Standardization of UDI interpretation across centers is essential for guideline adherence and multicenter research. AI-driven interpretation tools could reduce the training burden in settings where specialist expertise is limited. On the other hand, we face major challenges in training new colleagues to critically evaluate and question findings, especially in automated assessments (45).

### 4.2 Predictive risk stratification for upper urinary tract damage in SCI

The prevention of upper urinary tract (UUT) deterioration and renal failure is the paramount long-term goal of neuro-urological management in SCI. Currently, risk stratification depends on urodynamic findings, with high detrusor pressure (>40 cmH2O), DSD, low bladder compliance (<20 mL/cmH2O), and vesicoureteral reflux identified as key risk factors (46). However, early identification of patients at greatest risk, ideally before UUT damage occurs, requires the integration of multiple clinical, urodynamic, and neurological variables that AI is well-suited to process.

Based on predictors from the EMSCI models with using clinical parameters including lower extremity motor score (LEMS) obtained within 40 days of SCI, a multivariable logistic regression prediction model could be developed in 2023 to predict urodynamic risk factors for UUT damage within the first year (47). In 2025 a multivariate logistic regression analyses identified seven predictors of NLUTD incidence after SCI, validated in 115 SCI patients hospitalized between 2022 and 2024 with an overall accuracy of 97.14% (AUC 0.904, sensitivity 88.60%, specificity 82.50%). Predictor variables have been age, ASIA impairment grade, urinary culture results, CRP levels, voiding mode, pain scores, and sexual dysfunction (48). These works illustrate the feasibility of early, AI-assisted risk stratification in the acute phase of SCI, enabling proactive neuro-urological surveillance to be directed toward the highest-risk patients.

Automated extraction of features from pressure and volume recordings and fluoroscopic bladder images was used to predict the incident of hydronephrosis in patients with spina bifida at a median time of 1.6 to 2.49 years (24). Weaver et al. trained four machine learning models using data from videourodynamics on 354 patients in the training cohorts and 200 patients with spina bifida in the validation cohort. 89 patients in the training cohort and 71 patients in the validation cohort developed hydronephrosis. Especially the ensemble model constructed by the volume-pressure and fluoroscopic model reached a specificity of 97%.

ML has also been applied to predict multidrug-resistant UTI in brain and SCI patients. A dual-center validation study published in 2025 demonstrated that ML models could accurately identify SCI patients at risk for MDR-UTI, with important implications for antimicrobial stewardship in this population, who are among the highest-risk individuals for recurrent and complicated UTIs due to their dependence on catheterization and impaired immune surveillance of the urinary tract (49).

ML phenotyping of post-SCI NLUTD has been explored using the Neurogenic Bladder Research Group SCI registry, the largest prospective SCI registry in North America. Welk et al. applied unsupervised machine learning to identify distinct bladder phenotype clusters after SCI and assess their associations with urinary symptoms and QoL, demonstrating that ML-derived phenotypes captured clinically meaningful subgroups (especially the female cluster) not readily identified by conventional categorical classification (50).

### 4.3 Functional urology and neuromodulation

Sacral neuromodulation (SNM) is an established second-line treatment for overactive bladder, non-obstructive urinary retention, and fecal incontinence. While FDA approval for SNM is limited to non-neurogenic indications, a growing body of evidence supports its off-label use in carefully selected patients with NLUTD arising from Parkinson’s disease, incomplete SCI, MS, and spina bifida. AI is beginning to enhance SNM through the development of closed-loop, adaptive stimulation systems that dynamically adjust stimulation parameters based on real-time bladder pressure and voiding signals, rather than delivering fixed open-loop stimulation as current commercial devices do. Majerus et al. could demonstrate that closed-loop neuromodulation using only bladder pressure in the algorithm is feasible and well-tolerated, although further testings are needed to refine and validate this approach (32).

### 4.4 Bladder cancer surveillance in neurogenic bladder populations

A clinically important but underemphasized intersection of AI oncology tools and neuro-urology concerns bladder cancer surveillance in patients with chronic NLUTD. Long-term SCI patients carry a substantially elevated lifetime risk of bladder cancer compared with the general population (51). Current guidelines recommend that cystoscopic surveillance be considered in this population, although the evidence base for specific surveillance intervals and modalities remains limited. AI-enhanced cystoscopy (e.g. the CAIDS system referred to above) has the potential to improve the sensitivity of surveillance cystoscopy in this population, whose bladders frequently exhibit complex inflammatory changes, trabeculation, and diverticula that can confound lesion detection (52). Validation of AI cystoscopy tools in the neurogenic bladder population, where bladder anatomy and morphology differ substantially from non-neurogenic cohorts, is an important research priority.

### 4.5 Critical appraisal of the current evidence base

The applications summarized above should be interpreted with considerable caution. The reported accuracy figures – frequently exceeding 85–95% – derive almost exclusively from retrospective, single- or dual-center studies with modest sample sizes. Several of the risk-prediction models (for example, the seven-factor model validated in 115 patients, or the hydronephrosis model developed in a spina bifida cohort) report very high accuracy on internal or limited external validation, which raises a genuine concern regarding overfitting and optimistic performance estimates. Independent, prospective, multi-center validation against pre-specified reference standards is, at present, largely absent.

A further limitation is that almost all available studies report diagnostic or classification accuracy rather than patient-relevant outcomes. To date, no randomized or prospective study has demonstrated that AI-assisted urodynamic interpretation, risk stratification, or monitoring improves clinical endpoints such as renal preservation, prevention of autonomic dysreflexia, or QoL in NLUTD. Many models also function as ‘black boxes’ with limited interpretability, and their performance in the atypical, heterogeneous tracings characteristic of NLUTD– as opposed to the non-neurogenic cohorts on which several were partly trained – remains insufficiently established.

Finally, substantial barriers to clinical implementation persist beyond diagnostic performance. Few of the tools discussed have obtained regulatory clearance (CE marking or FDA approval) for use in neurogenic populations; questions of reimbursement, integration into existing clinical workflows, medico-legal liability for AI-informed decisions, and the need for continued clinician oversight are largely unresolved. Adaptive, continuous learning systems additionally challenge current regulatory frameworks, which were designed for static devices. These considerations temper the enthusiasm warranted by encouraging early results and frame the more forward-looking discussion that follows.

## 5 Emerging technologies under active development

### 5.1 Wearable biosensors, continuous bladder monitoring and drug delivery

One of the most frequently discussed emerging AI technology for patients with NLUTD is continuous, non-invasive bladder monitoring via wearable biosensor systems, although it should be emphasized that the supporting evidence is currently limited. The current standard for bladder monitoring in SCI, conventional urodynamic investigation in a specialist center, is episodic, invasive, and inaccessible outside tertiary care. The inability to continuously monitor bladder filling and pressure means special risks for individuals with SCI living in the community: First, bladder over-distension is the leading trigger of autonomic dysreflexia (AD), a potentially life-threatening hypertensive emergency unique to individuals with high-level SCI (T6 and above). Silent AD, occurring without pain sensation due to the sensory completeness of the injury, is a particular and frequent (53) danger.

Second, the repeated occurrence of high pressure in the bladder leads to significant secondary complications such as renal failure, reflux problems, irreversible morphological changes in the urinary tract and an increased likelihood of developing infections of the urogenital tract.

Stothers and Macnab presented findings from a feasibility study of a transcutaneous, near-infrared spectroscopy (NIRS)-based wearable optical bladder monitoring system in 66 individuals with SCI (54). The device tracked bladder volume and pressure through tissue optical density changes, simultaneously validated against conventional UDI. Optical density changes were successfully tracked across bladder filling stages: at volumes below 250 mL the lower sensor array detected signal changes first, while at volumes above 300 mL the upper array showed greater attenuation, enabling discrimination between filling stages. An end-user survey revealed that 90% of participants were willing to wear a bladder monitor even with mild movement restriction, with strong preference for smartphone integration and haptic alerts. This consumer-driven, patient-centered design approach is exemplary for new developments in NLUTD. It must be stressed, however, that this was a single feasibility study in a small cohort, without prospective clinical outcome data, and that these results require independent, larger-scale validation before any clinical application can be considered.

Complementary to optical sensing, a flexible strain sensor composed of gold-coated carbon nanotubes (AuCNT) embedded in Ecoflex substrate has been developed as a real-time bladder volume monitoring device, interfaced with a digital twin ‘Virtual Bladder’ model providing continuous visualization of bladder volume changes. Bioelectrical impedance analysis (BIA)-based wearable systems have demonstrated high correlation coefficients (96.7%) with bladder volume in preliminary studies (29). Implantable miniaturized pressure and volume sensors with wireless telemetry to external receivers represent a further development, particularly relevant for patients who cannot detect interoceptive bladder cues. In the long term, however, it is unlikely that measuring pressure inside the bladder using vesical sensors will be the decisive factor; rather, it will be the measurement of nerve activity between the bladder and the central nervous system.

Next to this development of wearable, implantable and intravesical soft bioelectronic devices for monitoring bladder conditions mechanisms for minimally invasive drug delivery are investigated. In the last years multiple different systems and devices have been designed like pumps (e.g. osmotic pumps, Alzet Pumps, DUROS osmotic pump), biodegradable implants, drug delivering hydrogels, T-/Y-shapes drug delivery devices and bio adhesive drug delivery systems like copolymers or magnetic nanoparticles with delivery by stimulation (55). Main problems are that the systems must be chemically stable, biofilm formation, the formation of stones or to provoke adverse immune reactions. They should mechanically align with bladder tissues to prevent irritation or erosion and at least, they should be interactable with bioelectronics to achieve the main goal of closed-loop therapeutic systems.

AI is the key to this, as it processes continuous data from different measurement systems and feedback-loops: raw sensor streams must be converted into clinically actionable information through algorithms that detect individualized filling thresholds, recognize precursors of dangerous detrusor pressure events, and generate patient alerts or transmit data to the clinical team. However, much like an insulin pump, the system would not only detect critical conditions and raise the alarm, but also initiate and monitor treatments in the form of controlled drug delivery.

Furthermore, AI-enabled digital health platforms could incorporate patient-reported outcomes, wearable sensor data, and electronic health record information to create a comprehensive longitudinal neuro-urological monitoring infrastructure, enabling individualized, data-driven follow-up that current in-person surveillance programs cannot deliver.

### 5.2 Closed-loop neuromodulation and AI-guided electrostimulation

Especially neuromodulation for NLUTD is moving toward closed-loop architectures in which the stimulation device continuously senses bladder or afferent nerve signals and autonomously adjusts stimulation parameters to maintain optimal bladder behavior. This approach would represent a qualitative advance over current commercial SNM devices, which deliver fixed stimulation patterns. AI serves as the interpretive and control layer in closed-loop systems: sensing algorithms classify bladder states (filling, impending contraction, emptying, pressure threshold breach), and control algorithms adjust stimulation amplitude, frequency, and timing accordingly.

In animal-models, an electrical neurostimulation approach by Majerus et al. demonstrated the ability to mimic reflexive urination control in SCI models, pointing toward the feasibility of biologically-inspired, AI-guided stimulation on architectures (32). This result has not yet been reproduced in humans.

At the same time, two clinical trials are investigating neuromodulation after acute traumatic spinal cord injuries with the aim to prevent NLUTD complications. The NCT03083366 trial - a multi-site, randomized clinical multi-center trial evaluating the effectiveness of early sacral neuromodulation (SNM) of the University of Utah – is analyzing the effectiveness of early sacral nerve stimulation in improving bladder-related complications and QoL. The TASCI-Study (NCT03965299) evaluate the use of transcutaneous tibial nerve stimulation (TTNS) within 40 days after acute SCI to prevent the occurrence of neurogenic detrusor overactivity by a nationwide, randomized, double-blind, sham-controlled trial conducted in Switzerland. Should the expected benefits occur, this would significantly enhance the clinical significance of neuromodulation. However, the significance of these two studies lies not only in the potential for early implementation of neuromodulation, but also in their role as a precursor to the integration of AI-driven predictive analytics into these trial frameworks to perform a real-time stratification of stimulation responders versus non-responders. Whether the effects of the stimulation could be enhanced by a closed-loop modulation has to be analyzed in the future.

### 5.3 AI in SCI rehabilitation: virtual assistants and multidisciplinary platforms

Early studies suggest that LLM-based virtual rehabilitation assistants can follow standardized clinical communication frameworks and answer patient queries about bladder management with accuracy above 90%. However, these findings derive from small, non-clinical evaluations, and the risk of hallucinated or unsafe advice in a life-safety context such as NLUTD is not yet adequately characterized. AI-driven rehabilitation planning platforms integrating IoT sensor data from wearables, electronic health record information, and patient-reported outcomes are under development, with particular attention to personalizing virtual physiotherapy assistants (VPAs) and exercise plans (35). This could promote adherence and effectiveness to rehabilitation programs (56). Furthermore, catheterization schedules, fluid intake recommendations, and medication timing for patients with NLUTD could be generated.

Digital twin technology, in which a patient-specific computational model of bladder structure and function is generated from imaging and urodynamic data, is an emerging platform for simulation-based treatment planning in NLUTD. By enabling in silico testing of different bladder management strategies before clinical implementation, digital twin models could reduce the trial-and-error period inherent in current NLUTD management and accelerate identification of optimal individualized therapy – though it should be noted that digital twin models for NLUTD currently remain conceptual, with no clinical validation yet reported in NLUTD populations (29).

### 5.4 AI-enhanced robotic surgery for neuro-urological procedures

Patients with NLUTD require surgical intervention, including augmentation cystoplasty, continent or incontinent urinary diversion, artificial urinary sphincter implantation, and bladder neck procedures. These are complex reconstructive operations typically performed in patients with challenging anatomy. AI-enhanced robotic surgical platforms (e.g. Da Vinci 5) with its AI-driven visualization and performance analytics features (57), offer particular value in reconstructive and prosthetic (58) surgery. Augmented reality overlays providing real-time anatomical guidance and AI-assisted tissue identification are under evaluation for cystoplasty and diversion procedures. For patients with SCI undergoing urinary reconstruction, the perioperative risks related to autonomic dysreflexia, pressure injury, and positioning complications create additional complexity that AI-driven pre-operative planning and intraoperative monitoring tools could address.

## 6 Opportunities and risks of AI in urology, with special reference to NLUTD

### 6.1 Opportunities

For the general urological population, AI offers improved diagnostic accuracy, reduced inter-observer variability, individualized treatment selection, and enhanced surgical precision. For patients with NLUTD specifically, additional opportunities, uniquely important ones, are: Automated, standardized urodynamic interpretation reduces the specialist dependency that currently limits access to guideline-compliant as well as individualized neuro-urological care. Predictive risk models for UUT deterioration enable proactive, individualized surveillance, reducing both the risk of renal damage and overtreatment. Continuous wearable bladder monitoring could fundamentally change the management of autonomic dysreflexia and prevent dangerous bladder over-distension events. Closed-loop neuromodulation could provide sustained, responsive treatment of neurogenic DO without the need for repeated invasive interventions. AI-powered rehabilitation platforms could extend specialist-level guidance to the community settings where the vast majority of persons with chronic SCI live.

AI also offers systemic health system benefits. In countries or regions where SCI survivors have limited access to specialist neuro-urological care, validated AI diagnostic tools could provide a critical bridge to safer bladder management – limited to the availability of medicines and medical aids.

### 6.2 Risks and limitations

The risks of AI in neuro-urology are both generic to medical AI and specific to the NLUTD population. Generic risks include algorithmic bias arising from non-representative training data, hallucination and overconfidence in LLM-based clinical tools, cybersecurity vulnerabilities in AI health platforms, and evolving regulatory pathways that may not adequately capture the adaptive nature of AI systems. Furthermore, AI may create false confidence or a wrong picture of truth (59). These risks are amplified in NLUTD because these patients are frequently underrepresented in AI training datasets, which have historically overrepresented ambulatory, non-disabled populations. An AI urodynamic interpretation system trained predominantly on non-neurogenic populations may perform poorly or unpredictably when applied to the complex urodynamic tracings characteristic for NLUTD. In addition, as historically, more males than females were affected by NLUTD due to SCI, sex- and gender-related differences may not be recognized accordingly.

Reliability is a heightened concern for NLUTD patients, whose bladder management decisions carry direct life-safety implications. An AI system that incorrectly interprets urodynamic tracings, misclassifies a pressure threshold, or fails to detect a precursor of autonomic dysreflexia could endanger the patient. Current AI validation studies in neuro-urology are predominantly retrospective, single-center, and limited in sample size, which constrains their generalizability.

Dependence and autonomy represent additional concerns. Patients with SCI already depend on assistive technologies for basic function; AI-driven bladder monitoring and management systems would add another layer of critical technological dependency. System failures, connectivity outages, device interactions or device malfunctions in AI-dependent bladder management have potentially severe consequences that do not apply to AI tools in less time-sensitive domains. Robust fail-safe design, offline fallback capabilities, and patient training in non-AI management alternatives are essential design requirements for any clinical AI system deployed in this population.

Ultimately, every person must always be viewed as a unique individual who may present conditions for which a standardized AI model has not been trained. A critical and individualized assessment, as well as ongoing and critical re-evaluation, must therefore always be the responsibility of the treatment team and must be considered in the individualized treatment plan, in consultation with the patient.

## 7 Ethical, moral, and societal implications

### 7.1 Disability rights, autonomy, and the human-AI relationship

The deployment of AI in the care of individuals with SCI and NLUTD must be approached through the lens of disability rights and the principles of the UN Convention on the Rights of Persons with Disabilities (CRPD). AI tools that improve function, independence, and access to care are consistent with these principles; AI systems that reduce patient autonomy, increase surveillance without consent, or substitute technological management for human relationship are not. Patients with SCI have frequently experienced healthcare systems that prioritize management efficiency over patient-centered outcomes and that underestimate the agency, expertise, and QoL of people with physical disabilities. The design of AI tools for this population must actively center patient perspectives and lived experience.

Informed consent for AI-assisted care in NLUTD is particularly complex. Patients with NLUTD engage in lifelong, continuous interaction with their urological care-givers, and AI tools embedded in that management, evolve over time as they learn from new data. Static, one-time consent processes are inadequate. Dynamic consent frameworks that provide patients with ongoing visibility into how AI systems are being updated, what data is being incorporated, and how AI-generated recommendations differ from those of their clinical team are necessary to preserve genuine informed consent. Aspects like the portable ethical and legal accountability at the clinician, data protection, bias and equity, transparency and explainability and supervision within approved governance frameworks must be clear (59).

### 7.2 Algorithmic bias and representation in NLUTD populations

Algorithmic bias is a systemic risk across medical AI, but the NLUTD population faces specific representational challenges. Traditionally, SCI predominantly affected young men following traumatic injury, but the global SCI population is diversifying, and NLUTD arising from neurological diseases including MS predominantly affects women, and demographics, ethology, and characteristics of SCI have significantly changed over time (60). AI systems trained on historical datasets may perform systematically differently across age, sex, injury etiology, lesion level, and completeness categories, with potentially dangerous consequences for risk stratification and treatment selection. Published SCI urodynamic datasets are substantially biased toward complete, traumatic, suprasacral injuries at tertiary centers in high-income countries, leaving incomplete injuries, cauda equina syndromes, non-traumatic etiologies, and patients in low-resource settings underrepresented.

The obligation to develop AI tools that perform equitably across the full spectrum of NLUTD must be built into the design process, not retrofitted after deployment. Federated learning frameworks, which enable training across multiple SCI centers internationally without transferring raw patient data, offer a practical pathway to building more diverse and representative training datasets. Post-deployment algorithmic auditing across demographic and injury subgroups should be mandatory, and transparent reporting of subgroup performance metrics should be required for regulatory approval of AI tools intended for neuro-urological use.

### 7.3 Socioeconomic access and global health equity

Most SCI survivors worldwide reside in low- and middle-income countries (LMICs), where access to neuro-urological specialist care, urodynamic equipment, medication, and evidence-based bladder management is limited. The consequences of inadequate NLUTD management in these settings can be devastating: renal failure, chronic UTI, and preventable mortality remain far more common than in high-income settings. AI has genuine potential to democratize access to neuro-urological expertise by enabling AI-augmented urodynamic interpretation at centers without specialized neuro-urologists, by supporting community-based bladder management through smartphone-linked wearable devices, and by providing AI-guided rehabilitation support in settings where specialist therapists are unavailable.

However, realizing this potential requires deliberate attention to infrastructure, connectivity, device cost, language accessibility, and local validation. AI tools validated on European or North American SCI populations may not generalize to populations in LMICs with different injury etiologies, comorbidity profiles, and healthcare contexts. The development of affordable, low-infrastructure AI diagnostic tools validated in LMIC populations, co-designed with local clinicians and patient communities, must become a priority for the global neuro-urological community. In addition, diagnostics alone will not aid in preventing secondary health issues. Prerequisite to an improved outcome is adequate access to treatment.

### 7.4 Data privacy, security, and the special sensitivity of NLUTD data

Continuous wearable bladder monitoring, remote urodynamic platforms, and other AI-enabled tools generate sensitive, granular data about the most intimate aspects of bodily function. For patients with SCI, whose bladder management is already an often stigmatized and socially complex dimension of disability experience, the aggregation and potential exposure of bladder monitoring data carries particular risks of privacy violation, stigma, and discrimination. Insurance companies, employers, and other parties gaining access to these data could use it to discriminate against individuals with SCI in insurance underwriting or employment.

The regulatory frameworks governing health data privacy, including the GDPR in Europe and HIPAA in the United States, were designed for episodic clinical data and are imperfectly adapted to the challenges of continuous, streaming wearable sensor data. New governance frameworks specifically addressing AI-generated continuous health monitoring data in vulnerable populations, including explicit limitations on secondary use, mandatory anonymization standards, and patient-controlled data access, are urgently needed. Neuro-urological societies, disability rights organizations, and patients themselves must be active participants in shaping these frameworks.

### 7.5 The urologist’s role: partnership, not substitution

The introduction of AI into neuro-urological care does not diminish the role of the urologist; it changes it. As AI assumes increasing responsibility for pattern recognition in urodynamic interpretation, risk stratification, and monitoring data analysis, the urologist’s most irreplaceable contributions become more, not less, salient: the integration of AI-generated information with the patient’s individual circumstances, preferences, and values; the communication of uncertainty and nuance; the advocacy for patients whose conditions render them particularly vulnerable to systematic under-recognition; and the exercise of final clinical and ethical judgment. The development of AI competencies in urological training, including the ability to critically evaluate AI-generated recommendations, recognize failure modes, and communicate AI-assisted findings to patients, is an emerging educational priority (45, 59, 61).

We advocate for a model of AI in neuro-urology as an empowering partner, amplifying the reach and precision of specialist expertise. AI should never substitute for it and should always subordinate to the judgment of the clinician and the values of the patient. This must be made particularly clear to insurance companies, large corporations and the political sphere, especially as otherwise a doctor can no longer be held legally, socially and ethically accountable. In particular for individuals with NLUTD, who depend on lifelong, trustworthy neuro-urological care, this partnership model is not merely preferable, it is ethically essential. Therefore, adequate training in neuro-urology remains essential to prevent complete dependence on diagnoses and treatment decisions made by AI-driven tools.

## 8 Perspective: a call for inclusive, patient-centered AI in neuro-urology

The evidence reviewed here supports a cautiously optimistic case for AI in neuro-urology across the five domains outlined above: automated urodynamic interpretation, predictive risk stratification, wearable monitoring, closed-loop neuromodulation, and LLM-based rehabilitation support. Importantly, most applications remain at an early, largely preclinical or proof-of-concept stage, and their eventual clinical value will depend on rigorous prospective validation.

But realizing these opportunities requires a concerted commitment to inclusion, validation, and equity that has not yet been adequately reflected in the neuro-urological AI literature. We call for four specific priorities. First, the deliberate inclusion of NLUTD populations, in their full demographic, etiological, and functional diversity, in the training and validation datasets of urological AI tools. Second, mandatory neurological subgroup performance reporting in AI validation studies. Third, co-design of AI tools for NLUTD with SCI survivors and individuals with NLUTD as active design partners, incorporating their lived experience and priorities into product development from the outset. And fourth, investment in affordable, low-infrastructure AI diagnostic tools validated for and with LMIC SCI populations, as a matter of global health justice.

The field of neuro-urology has a distinguished history of translating scientific advances into meaningful improvements in the lives of people with SCI: the systematic adoption of intermittent catheterization, urodynamically-guided management, and botulinum toxin therapy each dramatically reduced mortality and morbidity in this population. AI represents the next paradigm-shifting opportunity. The question is whether the neuro-urological community will rise to it with the rigor, inclusivity, and patient-centeredness it demands.

## 9 Conclusions

Artificial intelligence and robotics are transforming many areas of medicine at a rapid pace. Their influence across all areas of our lives is therefore undeniable and the question is no longer whether they will influence it, but to what extent. Currently, tech companies in particular are driving the development of these technologies. A critical approach that constantly questions their legality, data quality, ethical considerations and consequences is therefore unavoidable.

In urology AI holds transformative, specific potential for patients with NLUTD and SCI. Current applications include AI-augmented urodynamic interpretation, machine learning models for upper urinary tract risk prediction, AI-enhanced cystoscopy for bladder cancer surveillance, and AI-assisted robotic surgery. Emerging technologies of relevance to NLUTD include wearable transcutaneous bladder monitoring devices, closed-loop neuromodulation systems, digital twin bladder models, and LLM-based rehabilitation support platforms. The opportunities are substantial, encompassing improved diagnostic standardization, individualized risk stratification, real-time safety monitoring for autonomic dysreflexia, and expanded access to specialist-level neuro-urological guidance in resource-limited settings. The risks include algorithmic bias from non-representative training data, systemic dependency on technology with life-safety implications, data privacy vulnerabilities for sensitive continuous health data, and inequitable access between high- and low-income settings. The ethical dimensions demand dynamic consent frameworks, inclusive co-design with SCI survivors, equitable global development, and governance structures that protect the autonomy and dignity of individuals whose lives AI will increasingly shape. We advocate for AI as an empowering partner in neuro-urology, never as a replacement for the specialist expertise and humanistic commitment that patients with NLUTD deserve. We should therefore strive to design these tools within an interdisciplinary framework that incorporates ethical, economic, legal, and medically validated considerations. Additionally, mechanisms for control and intervention to prevent unchecked development must be considered at an early stage, as AI’s computational capabilities are, in many cases, far superior to our own.

## Data Availability

The original contributions presented in the study are included in the article/supplementary material. Further inquiries can be directed to the corresponding author.
