# Correction: Artificial intelligence and machine learning in neurogenic lower urinary tract dysfunction and spinal cord injury: current status, emerging technologies, and ethical perspectives

**DOI:** 10.3389/fruro.2026.1962187

**Published:** 2026-08-20

**Authors:** Frontiers Production Office

**Affiliations:** Frontiers Media, SA, Lausanne, Switzerland

**Keywords:** artificial intelligence, autonomic dysreflexia, clinical decision support, closed-loop neuromodulation, deep learning, detrusor-sphincter dyssynergia, digital twin, health equity

The title of this article was erroneously given as: “Artificial intelligence and machine learning in neurogenic tract dysfunction and spinal cord injury: current status, emerging technologies, and ethical perspectives”. The correct title of the article is “Artificial intelligence and machine learning in neurogenic lower urinary tract dysfunction and spinal cord injury: current status, emerging technologies, and ethical perspectives”.

The original version of this article has been updated.

